# The Membrane-Mediated Interaction of Liquid-Ordered Lipid Domains in the Presence of Amphipathic Peptides

**DOI:** 10.3390/membranes13100816

**Published:** 2023-09-28

**Authors:** Konstantin V. Pinigin, Sergey A. Akimov

**Affiliations:** Frumkin Institute of Physical Chemistry and Electrochemistry, Russian Academy of Sciences, 31/4 Leninskiy Prospekt, 119071 Moscow, Russia

**Keywords:** lipid membrane, theory of elasticity, liquid-ordered lipid domain, membrane-mediated interaction, domain interaction, raft coalescence, amphipathic peptide

## Abstract

The lipid membranes of living cells are composed of a large number of lipid types and can undergo phase separation with the formation of nanometer-scale liquid-ordered lipid domains, also called rafts. Raft coalescence, i.e., the fusion of lipid domains, is involved in important cell processes, such as signaling and trafficking. In this work, within the framework of the theory of elasticity of lipid membranes, we explore how amphipathic peptides adsorbed on lipid membranes may affect the domain–domain fusion processes. We show that the elastic deformations of lipid membranes drive amphipathic peptides to the boundary of lipid domains, which leads to an increase in the average energy barrier of the domain–domain fusion, even if the surface concentration of amphipathic peptides is low and the domain boundaries are only partially occupied by the peptides. This inhibition of the fusion of lipid domains may lead to negative side effects of using amphipathic peptides as antimicrobial agents.

## 1. Introduction

Lipid membranes are an important structural unit of living cells, serving as a semipermeable barrier surrounding cells and their organelles. Lipid membranes are also involved in functionally important processes such as endo- and exocytosis, vesicle trafficking, signal transduction, and the assembly of viruses, etc. [1,2]. It is known that the lipid composition of lipid membranes is heterogeneous: lipid molecules can be composed of different hydrophobic fatty acids and hydrophilic heads [3,4,5]. This heterogeneity may lead to phase separation into liquid-ordered and liquid-disordered phases [6,7,8,9,10]. In living cells, the liquid-ordered phase usually exists in the form of nanoscopic lipid domains, also called rafts [8,10,11]. These lipid domains represent inhomogeneities in the membrane plane and influence the lateral organization of biological membranes. In particular, membrane proteins can partition differently between the two phases [12,13,14,15]. Therefore, lipid domains, together with proteins, can form functional platforms in which certain processes are localized, for example, those associated with signal transduction [16,17,18]. Although nanoscopic lipid domains are difficult to observe directly, there is accumulating experimental evidence for the existence of these domains in vivo [19].

An important mechanism by which lipid domains are involved in cellular processes is raft coalescence, i.e., the fusion of small nanoscopic lipid domains into larger ones. Raft coalescence has been proposed to be involved in the immune response [13,20], signaling [16,21], and polarized sorting [22]. In addition, raft coalescence is indirectly observed in the endocytic recycling of plasma membrane proteins through raft-mediated budding [23,24]. In addition, experiments have shown the importance of raft coalescence in the assembly of integrin signaling complexes [25] and neuronal polarity determination [26].

Given the importance of raft coalescence in a variety of cell processes, its mis-regulation may have adverse effects on living cells. It is known, for example, that elevated levels of 7-dehydrocholesterol, which occur in Smith–Lemli–Opitz syndrome [27,28], significantly constrain the fusion of lipid domains [29]. Previously, within the framework of the theory of elasticity of lipid membranes, we showed that the adsorption of membrane-active antimicrobial amphipathic peptides at high concentrations onto lipid membranes with co-existing liquid-ordered and liquid-disordered phases leads to a significant increase in the domain–domain fusion energy barrier, due to the strong accumulation of the peptides at the domain boundaries, so that the boundaries are fully occupied by these peptides [30]. However, lower concentrations of amphipathic peptides have not been considered, and therefore, the natural question of to what extent low concentrations of amphipathic peptides affect the energy barrier of domain–domain fusion remains. Amphipathic peptides are currently considered to be promising antibiotics [31,32,33,34], and it is important to study the potential negative side effects of their use in antibacterial therapy. In this work, we generalize the results of Ref. [30] and consider the case of a low concentration of alpha-helical amphipathic peptides, assuming that the boundaries of the lipid domains are only partially occupied by the peptides.

## 2. Materials and Methods

### 2.1. Elastic Energy

In this work, within the theory of elasticity of lipid membranes, we consider the membrane-mediated interaction of liquid-ordered lipid domains in the presence of amphipathic peptides. We employ the results of the classical theory of elasticity adopted to lipid membranes and use the following quadratic elastic energy per unit area of the neutral surface of a lipid monolayer [35]:

(1) $$\begin{array}{l} w=\frac{1}{2}k_{m}{\left(\nabla \cdot n-J_{0}\right)}^{2}-\frac{1}{2}k_{m}J_{0}^{2}\\ +\frac{1}{2}k_{t}T_{}^{2}+k_{c}^{}T\cdot (\nabla \nabla \cdot n)+\frac{k_{gr}}{2}{\left(\nabla \nabla \cdot n\right)}^{2}\\ +\frac{1}{2}k_{A}^{}{\left(\alpha -\alpha_{0}\right)}^{2}-\frac{1}{2}k_{A}^{}\alpha_{0}^{2}-k_{c}^{}{\left(\nabla \alpha \right)}^{2}\\ +BT\cdot \nabla \alpha +C\nabla \alpha \cdot (\nabla \nabla \cdot n)+\sigma d(\Delta S). \end{array}$$

The neutral surface is defined as the surface with respect to which the deformations of bending and stretching compression are energetically decoupled; this surface lies approximately in the region of contact of the hydrophobic tails and hydrophilic heads of the lipid molecules [36]. In Equation (1), **n** is the vector field, called the director, of the unit vectors characterizing the average orientation of the lipid molecules; **T** is the tilt field, defined as $T\equiv \frac{n}{n\cdot N}-N$, where **N** is a unit normal vector to the monolayer neutral surface; α is the stretching compression deformation mode, i.e., the relative change in the area per lipid at the neutral surface; ∇ is the surface del operator; $k_{m}$ is the bending modulus; $k_{t}$ is the tilt modulus; $k_{A}$ is the stretching compression modulus; $k_{c}$, $k_{gr}$, *B*, and *C* are the elastic moduli corresponding to the deformation modes of T⋅(∇∇⋅n), ${\left(\nabla \nabla \cdot n\right)}_{}^{2}$, T⋅∇α, and ∇α⋅(∇∇⋅n), respectively; $J_{0}$ and $\alpha_{0}$ are the spontaneous curvature and spontaneous stretching compression, respectively; and σd(ΔS), where d(ΔS) is the local change in the surface area, is the energy term corresponding to the energy change due to the lateral tension of magnitude σ.

### 2.2. Elastic Parameters

The elastic parameters of the lipid domains and surrounding membrane are assumed to be different, as follows from experimental data. Let us use the indices “*d*” and “*s*” to denote the parameters of the lipid domains and surrounding membrane, respectively. First of all, the hydrophobic thickness is larger in the liquid-ordered phase and we set $h_{d}$ = 1.8 nm and $h_{s}$ = 1.3 nm [37,38]. For the monolayer bending modulus, which is also larger in lipid domains, we use the following values: $k_{ms}$ = 10 *k_B_T* and $k_{md}$ = 20 *k_B_T*, where *k_B_* is the Boltzmann constant and *T* ≈ 300 K is the absolute temperature [39,40,41,42,43]. The monolayer spontaneous curvatures are set to zero in both phases. As for the tilt modulus, $k_{t}$, we use the theoretically estimated value of 12 *k_B_T*/nm^2^ [44], which is close to experimental findings [45]. The results of Ref. [44] showed that the tilt modulus should not depend on the type of the phase, and, therefore, we will use the same value for the tilt modulus in both phases. The stretching compression modulus is also assumed to be equal in both phases, $k_{A}=k_{As}=k_{Ad}$ = 30 *k_B_T*/nm^2^ [42]. Although $k_{A}$ can be larger in the liquid-ordered phase, different values of $k_{A}$ in each phase are not of great importance for the elastic energy calculations, as stretching compression is a rather tough deformation mode. For $k_{c}$, $k_{gr}$, *B*, and *C* we use the theoretical estimates obtained in Ref. [35]: $k_{c}=-\frac{k_{t}h^{2}}{6}$, $k_{gr}=\frac{k_{t}h^{4}}{20}$, $B=-\frac{k_{t}h}{2}$, and $C=\frac{k_{t}h^{3}}{8}$, which leads to $k_{cs}$ = −3.4 *k*_B_*T*, $k_{cd}$ = −6.5 *k*_B_*T*, $k_{grs}$ = 1.7 *k*_B_*T*∙nm^2^, $k_{grd}$ = 6.3 *k*_B_*T*∙nm^2^, $B_{s}$ = −7.8 *k*_B_*T*/nm, $B_{d}$ = −10.8 *k*_B_*T*/nm, $C_{s}$ = 3.3 *k*_B_*T*∙nm, and $C_{d}$ = 8.7 *k*_B_*T*∙nm. The lateral tension per lipid monolayer is set to 0.1 mN/m ≈ 0.025 *k*_B_*T*/nm^2^ and is assumed to be equal in both phases [46,47], which leads to the spontaneous stretching compression, $\alpha_{0}\equiv \frac{\sigma }{k_{A}}$, equal to 8 × 10^−4^. The value of the lateral tension can depend on various circumstances, such as the cytoskeleton organization or cell motility, etc., but its exact value is not important for the results of this work. We also take into account the possibility of the misregistration of the opposing monolayers of the liquid-ordered domains. The energetic cost of this misregistration is assumed to be equal to 0.016 *k*_B_*T*/nm^2^ [48,49], which is given per unit misregistered area.

### 2.3. Parametrization of the System

We analyze the membrane deformations within the framework of a one-dimensional approach, according to which, the boundary of the lipid domains is considered to be a straight line, and the elastic energy is calculated per unit length along the domain boundary. This approach is numerically justified, provided that the proper effective interaction length is used to determine the absolute energy [50]. To describe the deformations of a lipid membrane, we introduce a Cartesian coordinate system, *xyz*, the *z*-axis of which is pointed upward and perpendicular to the membrane surface, while the *x*- and *y*-axes lie along the plane of the membrane, and the *y*-axis is parallel to the boundary of the lipid domains. The membrane is divided into regions of the liquid-ordered and liquid-disordered phases with the corresponding values of the elastic parameters. Using Equation (1), the elastic energy of each membrane region can be written as a sum of the elastic energies of the upper and lower monolayers:

(2) $$\begin{array}{l} w=\frac{k_{m}{}_{u}}{2}{\left(\frac{d}{dx}n_{u}+J_{u}\right)}^{2}-\frac{k_{mu}}{2}J_{u}^{2}+\frac{k_{t}}{2}{\left(n_{u}-\frac{d}{dx}H_{u}\right)}^{2}\\ +k_{cu}\left(n_{u}-\frac{d}{dx}H_{u}\right)\frac{d^{2}}{dx^{2}}n_{u}+k_{gru}{\left(\frac{d^{2}}{dx^{2}}n_{u}\right)}^{2}\\ +\frac{K_{a}}{2}{\left(-\frac{h_{u}{}^{2}\frac{d}{dx}n_{u}+2H_{u}-2M-2h_{u}}{2h_{u}}-\alpha_{0u}\right)}^{2}-\frac{K_{a}}{2}\alpha_{0u}^{2}\\ -\frac{B_{u}}{2h_{u}}\left(n_{u}-\frac{d}{dx}H_{u}\right)\left(h_{u}^{2}\frac{d^{2}}{dx^{2}}n_{u}+2\frac{d}{dx}H_{u}-2\frac{d}{dx}M\right)\\ -\frac{k_{cu}}{4h_{u}^{2}}{\left(h_{u}^{2}\frac{d^{2}}{dx^{2}}n_{u}+2\frac{d}{dx}H_{u}-2\frac{d}{dx}M\right)}^{2}\\ -\frac{C_{u}}{2h_{u}}\left(h_{u}^{2}\frac{d^{2}}{dx^{2}}n_{u}+2\frac{d}{dx}H_{u}-2\frac{d}{dx}M\right)\frac{d^{2}}{dx^{2}}n_{u}+\frac{\sigma }{2}{\left(\frac{d}{dx}H_{u}\right)}^{2}\\ +\frac{k_{m}{}_{l}}{2}{\left(\frac{d}{dx}n_{l}+J_{l}\right)}^{2}-\frac{k_{ml}}{2}J_{l}^{2}+\frac{k_{t}}{2}{\left(n_{l}+\frac{d}{dx}H_{l}\right)}^{2}\\ +k_{cl}\left(n_{l}+\frac{d}{dx}H_{l}\right)\frac{d^{2}}{dx^{2}}n_{l}+k_{grl}{\left(\frac{d^{2}}{dx^{2}}n_{l}\right)}^{2}\\ +\frac{K_{a}}{2}{\left(-\frac{h_{l}{}^{2}\frac{d}{dx}n_{l}-2H_{l}+2M-2h_{l}}{2h_{l}}-\alpha_{0l}\right)}^{2}-\frac{K_{a}}{2}\alpha_{0l}^{2}\\ -\frac{B_{l}}{2h_{l}}\left(n_{l}+\frac{d}{dx}H_{l}\right)\left(h_{l}^{2}\frac{d^{2}}{dx^{2}}n_{l}-2\frac{d}{dx}H_{l}+2\frac{d}{dx}M\right)\\ -\frac{k_{cl}}{4h_{l}^{2}}{\left(h_{l}^{2}\frac{d^{2}}{dx^{2}}n_{l}-2\frac{d}{dx}H_{l}+2\frac{d}{dx}M\right)}^{2}\\ -\frac{C_{l}}{2h_{l}}\left(h_{l}^{2}\frac{d^{2}}{dx^{2}}n_{l}-2\frac{d}{dx}H_{l}+2\frac{d}{dx}M\right)\frac{d^{2}}{dx^{2}}n_{l}+\frac{\sigma }{2}{\left(\frac{d}{dx}H_{l}\right)}^{2}, \end{array}$$

where the indices “*u*” and “*l*” correspond to the variables of the upper and lower monolayers, respectively; *H* and *M* are *z*-coordinates of the neutral surfaces of the monolayers and membrane mid-surface, respectively; and *h* is the hydrophobic thickness of the monolayers. To write Equation (2), the following relations are employed: $T_{u}=n_{u}-\frac{d}{dx}H_{u}$ and $T_{l}=n_{l}+\frac{d}{dx}H_{l}$ for the tilt field of the upper and lower monolayers, respectively, which follow from the definition of the tilt field; $\alpha_{u}=-\frac{h_{u}}{2}\frac{d}{dx}n_{u}-\frac{H_{u}}{h_{u}}+\frac{M}{h_{u}}+1$ and $\alpha_{b}=-\frac{h_{b}}{2}\frac{d}{dx}n_{b}+\frac{H_{b}}{h_{b}}-\frac{M}{h_{b}}+1$ for the stretching compression deformation mode of the upper and lower monolayers, respectively, which follow from the assumption of the volumetric incompressibility of lipid monolayers [35], according to experimental [51,52,53,54] and molecular dynamics [55,56,57] data; and $d(\Delta S)\approx \frac{1}{2}{\left(\frac{d}{dx}H\right)}^{2}$ for a local change in the surface area [35].

As the elastic energy density given by Equation (2) is quadratic, the corresponding Euler–Lagrange equations represent a linear system of ordinary differential equations. The system is too bulky to be presented here; however, it can be solved analytically. The general solutions for unknown functions, $n_{u}$, $n_{l}$, $H_{u}$, $H_{l}$, and *M*, have the form of $\sum_{i}C_{i}\exp\left(\lambda_{i}x\right)$, where $\lambda_{i}$ are known and $C_{i}$ are arbitrary constants. $C_{i}$ can be found analytically by substituting the solutions into the elastic energy and minimizing the resulting quadratic form, taking into account the following boundary conditions: the continuity of the director field, the continuity of the neutral surfaces of the monolayers, and the absence of the deformations far from the domain boundaries at *x* → ±∞. The solutions for $H_{u}$, $H_{l}$, and *M* provide the analytical expressions for the membrane shape, which will be used below for the illustrations of the membrane geometry.

### 2.4. Deformations Induced by Amphipathic Peptides

The amphipathicity of the peptides implies that approximately half of the side surface of the alpha-helix of the amphipathic peptides is hydrophilic, while the other half is hydrophobic [58]. Therefore, amphipathic peptides can be partially incorporated into lipid monolayers at the interface between the hydrophobic and hydrophilic parts of the lipid monolayers. This incorporation induces membrane deformations in the vicinity of the peptides [59,60]. To model these deformations, we consider amphipathic peptides as undeformable objects that impose certain boundary conditions on membrane deformations. We assume that there is a finite jump in the value of the director field at the boundary of the amphipathic peptides:

(3) $$\Delta n_{x}=n_{2x}-n_{1x}=\frac{\Delta L}{\sqrt{{\left(\Delta L/2\right)}^{2}+{\left(h/2\right)}^{2}}},$$

where ΔL is the diameter of the peptide alpha-helix; h is the hydrophobic thickness of the lipid monolayer adjacent to the peptides; and $n_{1x}$ and $n_{2x}$ are the projections of the director field onto the *x*-axis at the left and right boundaries of the peptides, respectively. Equation (3) is the estimate of the director jump based on the geometric considerations: it is assumed that the boundary director is directed towards the center of the hydrophobic region of the monolayer in which the peptides are located. These geometric considerations are also consistent with the membrane thinning induced by amphipathic peptides observed in experiments and molecular dynamics [61,62], which we observe within our model in the vicinity of the amphipathic peptides. We set ΔL to 1.3 nm, which is a typical alpha-helix diameter [63,64]. Note that $\Delta n_{x}$ is different in the liquid-ordered and liquid-disordered phases, as h is larger in the liquid-ordered phase. Therefore, if the peptides are located at the interphase between the two phases, we set $\Delta n_{x}=\delta \Delta n_{x}{}_{s}+\left(1-\delta \right)\Delta n_{x}{}_{d}$, where $\Delta n_{x}{}_{s}$ and $\Delta n_{x}{}_{d}$ are the director jumps in the liquid-disordered and liquid-ordered phases, respectively, and δ is the fraction of the peptide located in the liquid-disordered phase. In addition, we take into account the possibility of the rotation of the amphipathic peptides around the axis of the alpha-helix:

(4) $$H_{u}(X_{0}+(\Delta L/2))-H_{u}(X_{0}-(\Delta L/2))=\Delta L\;(n_{1x}+n_{2x})/2,$$

where $X_{0}$ is the *x*-coordinate of the peptide center and $H_{u}\left(x\right)$ is the shape of the neutral surface of the monolayer adjacent to the peptides.

It is known that amphipathic peptides usually induce membrane softening [65,66,67], i.e., a decrease in the bending modulus of a lipid membrane, which might be caused by the composition–curvature coupling effect [68]. This softening is a statistical effect relevant for large membrane patches, upon which many amphipathic peptides are adsorbed. In this work, the scale of the considered system is several nanometers and only one or two amphipathic peptides are considered. Therefore, we assume that the elastic parameters of the lipid monolayers in contact with the amphipathic peptides are the same as in the absence of amphipathic peptides.

## 3. Results

### 3.1. Interaction of Amphipathic Peptides with Lipid Domains

**Figure 1:**
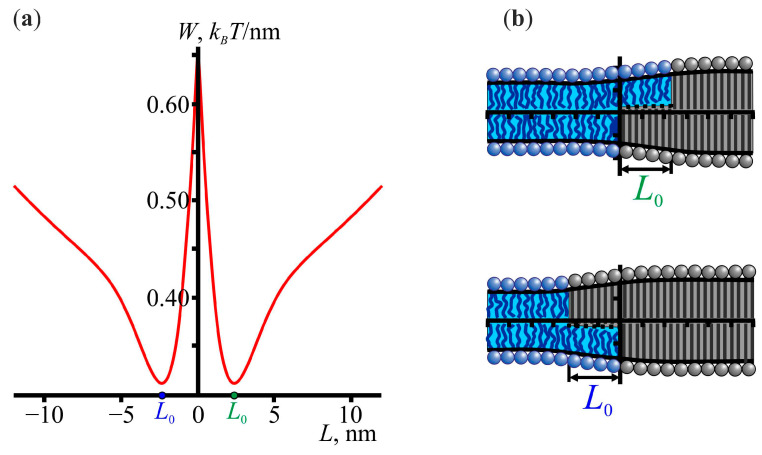
(**a**) The dependence of the elastic energy on the relative shift *L* of the monolayers of lipid domains. (**b**) The theoretically calculated membrane shapes at the values of *L* corresponding to the minima of the elastic energy. Lipid domains are shown in grey; the surrounding membrane is shown in blue; lipid heads and tails are drawn for illustrative purposes; the black curves represent the shapes of the neutral surfaces; and the dotted curves represent the shape of the mid-surface.

The interaction of amphipathic peptides with lipid domains occurs mainly at the domain boundaries. The mismatch between the hydrophobic thickness of the lipid domains and the surrounding membrane leads to elastic deformations at their boundary. These deformations can overlap with the deformations induced by amphipathic peptides, leading to an effective membrane-mediated interaction between the lipid domains and the peptides. To consider this interaction, it is important to take into account that the structure of the boundary of lipid domains is not symmetric. In Ref. [69], it was shown that the monolayers of lipid domains are slightly shifted relative to each other, which follows from the minimization of elastic energy. This shift has also been observed in molecular dynamics simulations [38,70,71]. Figure 1 shows the dependence of the elastic energy on the size *L* of the relative shift between the opposing monolayers.

For the chosen set of elastic parameters, the elastic energy was minimal at |*L*| ≈ 2.3 nm. The value of the elastic energy at the minimum points was ≈0.3 *k_B_T*/nm. Note that this energy was given per unit length along the domain boundary and thus represented the elastic energy contribution to the line tension of the domain boundary. In general, the chemical interactions of the lipid molecules at the domain boundary also contribute to the line tension. We assumed that the latter contribution had some constant value and therefore did not affect the elastic energy barrier of the domain–domain fusion, which will be calculated below.

**Figure 2:**
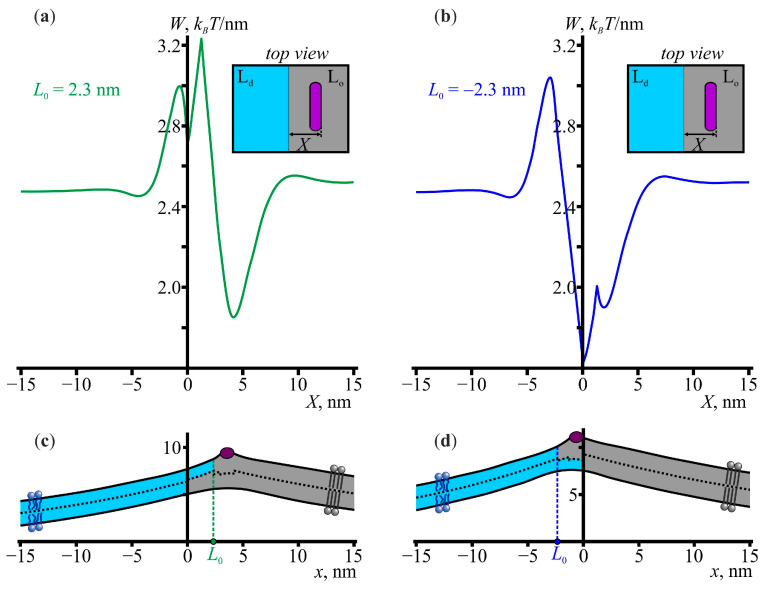
The membrane-mediated interaction between amphipathic peptides and liquid-ordered lipid domains. (**a**,**b**) The elastic energy profiles as a function of the coordinate *X* of the right boundary of the amphipathic peptide at the relative shift of the liquid-ordered monolayers equal to (**a**) *L*_0_ = 2.3 nm and (**b**) *L*_0_ = −2.3 nm. The insets show the top view of the membrane. The liquid-disordered (L_d_) and liquid-ordered (L_o_) phases are shown in blue and grey, respectively; amphipathic peptides are shown in purple. (**c**,**d**) The theoretically calculated membrane shapes at the values of *X* corresponding to the global energy minima of the energy profiles shown in panels (**a**,**b**).

Notice that *L* could be either positive (*L* ≈ +2.3 nm) or negative (*L* ≈ −2.3 nm), depending on the relative arrangement of the monolayers. Consequently, considering the interaction of the amphipathic peptides with the lipid domains, we should distinguish between the cases of positive *L* and negative *L*. The corresponding elastic energy profiles are shown in Figure 2. There was a rather deep energy well near the domain boundary, i.e., the location of the peptides near the domain boundaries was energetically favorable. It should be noted that the corresponding elastic energy profiles could be plotted at different values of *L*, and it turned out that the global energy minima corresponded to *L* ≈ +0.9 nm and *L ≈* −2.4 nm, which were different from *L* ≈ +2.3 nm and *L* ≈ −2.3 nm, respectively, in the case of no peptides. The locations, *X*, of the right boundary of the peptide at the global minima were *X* ≈ 2.7 nm and *X* = 0 nm at *L* > 0 and *L* < 0, respectively.

### 3.2. Domain–Domain Fusion Energy Barrier at a Low Concentration of Amphipathic Peptides

**Figure 3:**
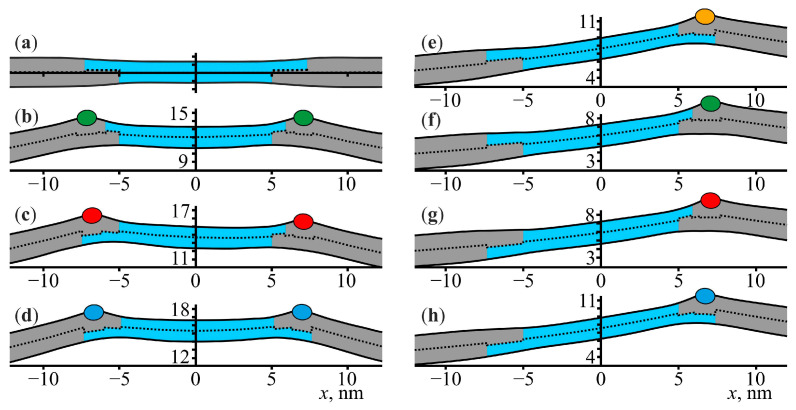
The theoretically calculated shapes of the lipid membrane with coexisting liquid-ordered (grey) and liquid-disordered (blue) phases at different amounts of amphipathic peptides (colored ellipses). (**a**) No amphipathic peptides. (**b**–**d**) Two amphipathic peptides. (**e**–**h**) One amphipathic peptide. The membrane shapes are calculated for different configurations of the domain boundaries, corresponding to different relative shifts of the opposing monolayers of the lipid domains. Only those shapes are shown which are not equivalent in terms of the elastic energy. The distance between the domains is equal to 10 nm in all panels.

The membrane elastic deformations at the boundaries of lipid domains are independent when these domains are located far from each other. However, at a sufficiently small distance between the domains, these deformations overlap, which leads to an effective lateral interaction between the domains. This interaction was previously considered in Refs. [29,72], and it was shown that there was an energy barrier of approximately 0.1 *k_B_T*/nm that was necessary to overcome to bring two domains into close contact. In Ref. [30], we considered the membrane-mediated interaction of lipid domains in the presence of amphipathic peptides under the assumption that the boundaries of lipid domains are completely occupied by these peptides. The corresponding configurations of the boundaries are shown in Figure 3b–d. The results showed that the domain–domain fusion energy barrier significantly increases due to the presence of amphipathic peptides at the domain boundaries.

Now, we consider a more general case of the partial occupation of domain boundaries by peptides. To model this within the one-dimensional approach, we considered the case of when the boundary of only one of the domains was occupied by an amphipathic peptide. We assumed that the peptide was located at the boundary of the right domain (see Figure 3e–h) and calculated the elastic energy as a function of the distance between the domains. The choice between the left or right domain for the peptide location was arbitrary: due to random diffusion, the peptide would eventually end up in the potential well at the boundary of the right or left domain, but, due to the symmetry of the system, the domain–domain fusion energy barrier did not depend on whether the considered peptide was located at the boundary of the right or left domain. To accomplish this, we considered all four possible configurations of the domain boundaries. At a given distance *D* between the domains, we minimized the elastic energy with respect to the peptide position and relative shifts *L* of the monolayers of the left and right lipid domains. Note that, in the case of one amphipathic peptide, four different configurations of the domain boundaries should be considered (Figure 3e–h), while in the case of two amphipathic peptides, only three configurations were possible (Figure 3b–d), as the elastic energy of the configuration (c) in Figure 3 did not change upon reflection in the plane *x* = 0. Additionally, the elastic energy in the case of no peptides (Figure 3a) did not depend on the particular configuration of the domain boundaries [30].

**Figure 4:**
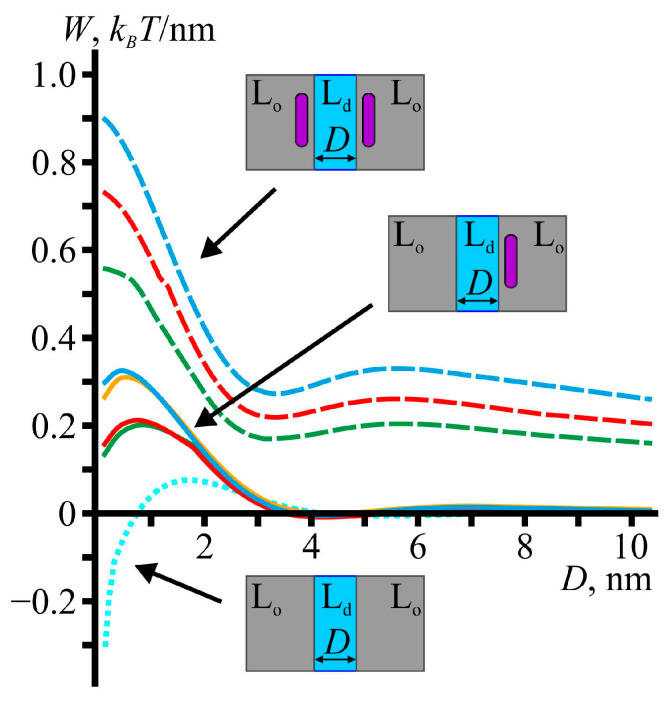
The elastic energy of the interaction of liquid-ordered lipid domains as a function of the distance between the domains. The dotted cyan curve shows the interaction energy between domains in the case of no amphipathic peptides. The solid curves correspond to the case of one amphipathic peptide located at the boundary of one of the domains. The dashed curves correspond to the case when both domain boundaries are occupied by amphipathic peptides. The colors of the curves correspond to the colors of amphipathic peptides in Figure 3, depending on the configuration of the domain boundaries. The insets illustrate the top view of the considered cases. The liquid-ordered lipid domains (L_o_) are shown in grey, the liquid-disordered phase (L_d_) is shown in blue, and amphipathic peptides are shown in purple.

The results are shown in Figure 4. The dashed curves represent the dependence of the elastic energy of the membrane on the distance *D* between the lipid domains, in the case when both domain boundaries were occupied by the peptides. The dotted cyan curve corresponds to the case of no peptides [30]. The remaining solid curves correspond to the case when only one domain boundary was occupied by the amphipathic peptide. The colors of the solid curves correspond to the configurations and colors of the peptides shown in Figure 3e–h. All the curves represent the elastic energy minus the energy at an infinite distance between the domains, D=+∞. It can be seen that the energy barriers in the case of one amphipathic peptide had intermediate values between the energy barriers in the case of no peptides and two peptides. In the case of one amphipathic peptide, the energy barrier was slightly higher when the domain with the peptide was larger in the upper leaflet. In general, the energy barrier in the case of one amphipathic peptide was approximately 0.2–0.3 *k_B_T*/nm, which was about 3–4 times larger than the energy barrier of approximately 0.08 *k_B_T*/nm in the case of no peptides. The values of 0.2–0.3 *k_B_T*/nm were, however, smaller than the energy barrier in the case of two peptides, which ranged approximately from 0.6 to 0.9 *k_B_T*/nm.

## 4. Discussion

In this work, within the classical theory of elasticity of lipid membranes, we considered the membrane-mediated interaction of liquid-ordered lipid domains, the boundaries of which were partially occupied by amphipathic peptides. The obtained results generalized the study of Ref. [30], in which the domain–domain fusion energy barrier was calculated under the assumption that the domain boundaries were completely occupied by amphipathic peptides. In Ref. [30], it was shown that the amphipathic peptides significantly increased the fusion energy barrier of the lipid domains from approximately 0.1 *k_B_T*/nm in the case of no peptides to approximately 0.6–0.9 *k_B_T*/nm in the case when the domain boundaries were completely occupied by the peptides. The results of the present study show that the domain–domain fusion energy barrier increased not only when the boundaries of lipid domains were completely occupied by amphipathic peptides, but also when the domain boundaries were only partially occupied by these peptides.

We employed a one-dimensional approach to the calculation of the domain–domain fusion energy barrier. Within this approach, it was assumed that the domain boundary represented a straight line and the energy of the system was calculated per unit length along the domain boundary. This approach was justified by the small characteristic length, λ ≈ 1 nm, of the spatial propagation of the amplitude of the elastic deformations of the lipid membranes [30]. This one-dimensional approach can be also successfully applied to the analysis of the membrane-mediated interaction between membrane inclusions [50]. To obtain the absolute value of the elastic energy, the energy per unit length of the domain boundary should be multiplied by the effective length of the domain–domain interaction, which can be estimated as $2\sqrt{2\lambda R}$ [30,73], where *R* is the domain radius.

As can be seen from Figure 2, after adsorption, the amphipathic peptides were localized mainly at the boundaries of the lipid domains. Therefore, a natural question to consider is how the amphipathic peptides affected the energy barrier of the domain–domain fusion. Within the one-dimensional approach, the membrane-mediated interaction of the lipid domains with different amounts of amphipathic peptides at their boundaries could be modeled by considering three different cases: (1) no peptides at the boundaries of the lipid domains, (2) one peptide at the boundary of one of the domains and no peptide at the boundary of the other domain, and (3) two peptides with one peptide at each boundary. The first and third cases were previously considered in Ref. [30], where it was shown that the energy barrier of the domain–domain fusion increased from approximately 0.1 *k_B_T*/nm in the case of no peptides to 0.6–0.9 *k_B_T*/nm when both domain boundaries were occupied by the peptides. In this work, we additionally considered the second case, when only one boundary was occupied by a peptide. The energy barrier, in this case, turned out to be approximately 0.2–0.3 *k_B_T*/nm, which was an intermediate value between the energy barriers for the cases of no peptides and two peptides. Among the considered configurations of the domain boundaries, the most physiologically relevant ones were those in which the lipid domains were larger in the upper leaflet, as the outer leaflet of plasma membranes is usually more enriched with saturated lipids than the cytosolic leaflet [74,75]. For this configuration, the energy barriers of the domain–domain fusion were approximately 0.9 and 0.3 *k_B_T*/nm in the cases of two peptides and one peptide, respectively (see Figure 4).

It was pointed out in Ref. [30] that the observed increase in the energy barrier was largely caused by the peptide–peptide repulsion at a close distance between the peptides, which followed from the elastic energy of the membrane-mediated interaction between the amphipathic peptides [30,50,76]. In this sense, it is not obvious whether the domain–domain fusion energy barrier would increase if only one peptide at the boundary of one of the domains is considered. Notice, however, that, as followed from the analysis of the membrane-mediated interaction between the amphipathic peptides and the lipid domains (Figure 2), there was a nonzero energy barrier of approximately 0.6–0.8 *k_B_T*/nm that the peptides had to overcome to pass from the liquid-disordered phase to the liquid-ordered phase. Therefore, the amphipathic peptides experienced repulsion from the domain boundaries when they were located in the liquid-disordered phase near the domain boundaries, and it was this repulsion that led to the increase in the domain–domain fusion energy barrier when the domain boundaries were partially occupied by the peptides. This means that, at a low surface concentration of amphipathic peptides, the increase in the energy barrier is mainly due to the peptide–domain repulsion, and not the peptide–peptide repulsion, the probability of which is small at a low concentration of amphipathic peptides. Thus, the energy barrier of the domain–domain fusion increases faster with an increase in the surface concentration of the amphipathic peptides compared to if only the peptide–peptide repulsion is taken into account.

The number of amphipathic peptides at the boundaries of the lipid domains depended on the concentration of the peptides on the surface of the membrane. Let us estimate the peptide concertation, *P*/*L* (the number of peptides per lipid), at which the boundary of the lipid domains appeared to be fully occupied by the peptides. If the area fraction of the lipid domains is $\varphi_{d}$ and the total membrane area is $A_{0}$, then the total number of lipid domains of a mean radius *R* is $N_{d}=\frac{\varphi_{d}A_{0}}{\pi R^{2}}$ and the total length of the domain boundaries is $2\pi RN_{d}$. Therefore, the total number of amphipathic peptides required to completely occupy the boundaries of the lipid domains can be estimated as $\frac{2\pi RN_{d}}{L_{p}}$, where $L_{p}$ is the length of the peptide alpha-helix. In this estimate, it was implicitly assumed that the energy well corresponding to the location of the amphipathic peptides at the boundaries of the lipid domains was high enough to neglect the redistribution of the peptides over the membrane surface. In fact, as follows from Figure 2, this energy well was approximately 0.8 *k_B_T*/nm. For a peptide of length $L_{p}$, the absolute value of this energy well was ≈0.8 × 1.3*L_p_*, where 1.3*L_p_* is the effective length of the membrane deformations induced by a peptide [50]. For a typical amphipathic peptide, such as magainin (*L_p_* ≈ 3.5 nm [77]), the energy well was approximately 3.5 *k_B_T*, which is seven times higher than the energy of the thermal motion per degree of freedom, 0.5 *k_B_T*. Thus, it can be assumed that the amphipathic peptides were located mainly at the boundaries of the lipid domains. Within this assumption, the ratio of the number of amphipathic peptides to the total number of lipids, $\frac{A_{0}}{a_{L}}$, where $a_{L}$ is the mean area per lipid, is *P*/*L* = $\frac{2a_{L}\varphi_{d}}{L_{p}R}$. For a standard, raft-forming mixture of dioleoylphosphatidylcholine, dipalmitoylphosphatidylcholine, and cholesterol in approximately equal concentrations, the experimental value of the area fraction, $\varphi_{d}$, of the liquid-ordered phase depends on temperature, but the mean value can be estimated as ≈30% [78]. The typical value of the area per lipid is $a_{L}$ ≈ 0.6 nm^2^ [79]. The peptide length $L_{p}$ depends on the peptide structure. As an example, let us consider magainin: $L_{p}$ ≈ 3.5 nm [77]. The mean radius of the lipid domains, *R*, may depend on the lipid composition, but in practically relevant cases, *R* is usually less than approximately 100 nm [80]. For these parameter values, *P*/*L* ≈ 1/50, 1/500, and 1/1000 if *R* = 5, 50, or 100 nm, respectively. These concentrations are lower than the critical pore-forming concentration (*P*/*L* ≈ 1/30) of magainin [59]. As the results of this work showed, even if the boundaries of lipid domains are not completely occupied by peptides, the average domain–domain fusion energy barrier nevertheless increases. Therefore, amphipathic peptides can affect the domain–domain fusion energy barrier at concentrations much lower than the critical concentration of the pore formation by the peptides.

Increasing experimental evidence suggests the importance of the participation of nanoscopic liquid-ordered lipid domains, or so-called rafts, in signal transduction and trafficking [19]. It has been proposed that important cellular processes, such as immune responses [13,20], signaling [16,21], and polarized sorting [22], require the coalescence of rafts, i.e., the fusion of small, nanometer-scale domains to larger ones. There is also experimental evidence that has shown the involvement of raft coalescence in neural polarity determination [26], the assembly of integrin signaling complexes [25], and raft-dependent endocytic sorting [23,24]. It is known that the mis-regulation of raft coalescence is associated with Smith–Lemli–Opitz syndrome [27,28,29]. The results of this work showed that amphipathic peptides, which are currently considered to be perspective antimicrobial drugs, may have a negative impact on the normal regulation of the processes associated with raft coalescence. Amphipathic peptides usually have a positive charge that determines their selectivity to the negatively charged membranes of bacteria, in which the amphipathic peptides form pores and thereby kill pathogens [81,82]. However, the amphipathic properties of these peptides can also lead to the undesirable adsorption of amphipathic peptides to the normal cells of a human organism. As a measure of toxicity, the hemolytic activity of these amphipathic peptides is usually considered [58]. In this work, another possible mechanism of the toxicity of amphipathic peptides was considered, which involved the mis-regulation of raft coalescence.

## 5. Conclusions

In this work, we showed that amphipathic peptides impeded the fusion of liquid-ordered lipid domains, even at a low concentration of amphipathic peptides on the surfaces of the lipid membranes. This occurred because the elastic deformations of the lipid membranes drove the peptides towards the boundaries of the lipid domains upon the adsorption of the peptides on the lipid membranes with coexisting liquid-ordered and liquid-disordered phases, which led to an increase in the energy barrier of the domain–domain fusion. As was shown in this work, this energy barrier increased even if the boundaries of the lipid domains were only partially occupied by the peptides. Thus, even at a low concentration of amphipathic peptides, which is much lower than the critical concertation for the pore formation by peptides, the mis-regulation of raft coalescence, which is involved in important cellular processes, could occur. Thus, the undesirable binding of amphipathic peptides to normal human cells may adversely affect the lateral organization of lipid membranes and lead to possible negative side effects when using amphipathic peptides as antimicrobial agents.

## Data Availability

Data available on request.

## References

[1] Yeagle P.L. (2016). The Membranes of Cells.

[2] Nelson L.D., Cox M.M. (2004). Lehninger Principles of Biochemistry.

[3] Van Meer G., Voelker D.R., Feigenson G.W. (2008). Membrane lipids: Where they are and how they behave. Nat. Rev. Mol. Cell Biol..

[4] Levental I., Veatch S.L. (2016). The continuing mystery of lipid rafts. J. Mol. Biol..

[5] Levental K.R., Surma M.A., Skinkle A.D., Lorent J.H., Zhou Y., Klose C., Chang J.T., Hancock J.F., Levental I. (2017). ω-3 polyunsaturated fatty acids direct differentiation of the membrane phenotype in mesenchymal stem cells to potentiate osteogenesis. Sci. Adv..

[6] Samsonov A.V., Mihalyov I., Cohen F.S. (2001). Characterization of Cholesterol-Sphingomyelin Domains and Their Dynamics in Bilayer Membranes. Biophys. J..

[7] Pralle A., Keller P., Florin E.L., Simons K., Hörber J.H. (2000). Sphingolipid–cholesterol rafts diffuse as small entities in the plasma membrane of mammalian cells. J. Cell Biol..

[8] Owen D.M., Williamson D.J., Magenau A., Gaus K. (2012). Sub-resolution lipid domains exist in the plasma membrane and regulate protein diffusion and distribution. Nat. Commun..

[9] Ayuyan A.G., Cohen F.S. (2008). Raft composition at physiological temperature and pH in the absence of detergents. Biophys. J..

[10] Frisz J.F., Lou K., Klitzing H.A., Hanafin W.P., Lizunov V., Wilson R.L., Carpenter K.J., Kim R., Hutcheon I.D., Zimmerberg J. (2013). Direct chemical evidence for sphingolipid domains in the plasma membranes of fibroblasts. Proc. Natl. Acad. Sci. USA.

[11] Nickels J.D., Chatterjee S., Stanley C.B., Qian S., Cheng X., Myles D.A.A., Standaert R.F., Elkins J.G., Katsaras J. (2017). The in vivo structure of biological membranes and evidence for lipid domains. PLoS Biol..

[12] Lorent J.H., Diaz-Rohrer B., Lin X., Spring K., Gorfe A.A., Levental K.R., Levental I. (2017). Structural determinants and functional consequences of protein affinity for membrane rafts. Nat. Commun..

[13] Levental I., Grzybek M., Simons K. (2010). Greasing their way: Lipid modifications determine protein association with membrane rafts. Biochemistry.

[14] Pinigin K.V., Kondrashov O.V., Jiménez-Munguía I., Alexandrova V.V., Batishchev O.V., Galimzyanov T.R., Akimov S.A. (2020). Elastic deformations mediate interaction of the raft boundary with membrane inclusions leading to their effective lateral sorting. Sci. Rep..

[15] Kondrashov O.V., Pinigin K.V., Akimov S.A. (2021). Characteristic lengths of transmembrane peptides controlling their tilt and lateral distribution between membrane domains. Phys. Rev. E.

[16] Simons K., Toomre D. (2000). Lipid rafts and signal transduction. Nat. Rev. Mol. Cell Biol..

[17] Simons K., Sampaio J.L. (2011). Membrane organization and lipid rafts. Cold Spring Harb. Perspect. Biol..

[18] Sezgin E., Levental I., Mayor S., Eggeling C. (2017). The mystery of membrane organization: Composition, regulation and roles of lipid rafts. Nat. Rev. Mol. Cell Biol..

[19] Levental I., Levental K.R., Heberle F.A. (2020). Lipid rafts: Controversies resolved, mysteries remain. Trends Cell Biol..

[20] Varshney P., Yadav V., Saini N. (2016). Lipid rafts in immune signalling: Current progress and future perspective. Immunology.

[21] Mollinedo F., Gajate C. (2015). Lipid rafts as major platforms for signaling regulation in cancer. Adv. Biol. Regul..

[22] Schuck S., Simons K. (2004). Polarized sorting in epithelial cells: Raft clustering and the biogenesis of the apical membrane. J. Cell Sci..

[23] Diaz-Rohrer B.B., Levental K.R., Simons K., Levental I. (2014). Membrane raft association is a determinant of plasma membrane localization. Proc. Natl. Acad. Sci. USA.

[24] Diaz-Rohrer B., Castello-Serrano I., Chan S.H., Wang H.Y., Shurer C.R., Levental K.R., Levental I. (2023). Rab3 mediates a pathway for endocytic sorting and plasma membrane recycling of ordered microdomains. Proc. Natl. Acad. Sci. USA.

[25] Altrock E., Muth C.A., Klein G., Spatz J.P., Lee-Thedieck C. (2012). The significance of integrin ligand nanopatterning on lipid raft clustering in hematopoietic stem cells. Biomaterials.

[26] Honda A., Ito Y., Takahashi-Niki K., Matsushita N., Nozumi M., Tabata H., Takeuchi K. (2017). Extracellular signals induce glycoprotein M6a clustering of lipid rafts and associated signaling molecules. J. Neurosci..

[27] Smith D.W., Lemli L., Opitz J.M. (1964). A newly recognized syndromeof multiple congenital anomalies. J. Pediatr..

[28] Porter F.D. (2008). Smith–Lemli–Opitz syndrome: Pathogenesis, diagnosis and management. Eur. J. Hum. Genet..

[29] Staneva G., Osipenko D.S., Galimzyanov T.R., Pavlov K.V., Akimov S.A. (2016). Metabolic precursor of cholesterol causes formation of chained aggregates of liquid-ordered domains. Langmuir.

[30] Pinigin K.V., Galimzyanov T.R., Akimov S.A. (2021). Amphipathic peptides impede lipid domain fusion in phase-separated membranes. Membranes.

[31] Fjell C.D., Hiss J.A., Hancock R.E., Schneider G. (2012). Designing antimicrobial peptides: Form follows function. Nat. Rev. Drug Discov..

[32] Da Costa J.P., Cova M., Ferreira R., Vitorino R. (2015). Antimicrobial peptides: An alternative for innovative medicines?. Appl. Microbiol. Biotechnol..

[33] Bahar A.A., Ren D. (2013). Antimicrobial peptides. Pharmaceuticals.

[34] Guha S., Ghimire J., Wu E., Wimley W.C. (2019). Mechanistic landscape of membrane-permeabilizing peptides. Chem. Rev..

[35] Pinigin K.V., Kuzmin P.I., Akimov S.A., Galimzyanov T.R. (2020). Additional contributions to elastic energy of lipid membranes: Tilt-curvature coupling and curvature gradient. Phys. Rev. E.

[36] Leikin S., Kozlov M.M., Fuller N.L., Rand R.P. (1996). Measured effects of diacylglycerol on structural and elastic properties of phospholipid membranes. Biophys. J..

[37] Rinia H.A., Snel M.M., van der Eerden J.P., de Kruijff B. (2001). Visualizing detergent resistant domains in model membranes with atomic force microscopy. Febs Lett..

[38] Risselada H.J., Marrink S.J. (2008). The molecular face of lipid rafts in model membranes. Proc. Natl. Acad. Sci. USA.

[39] Evans E., Rawicz W. (1990). Entropy-driven tension and bending elasticity in condensed-fluid membranes. Phys. Rev. Lett..

[40] Pan J., Tristram-Nagle S., Nagle J.F. (2009). Effect of cholesterol on structural and mechanical properties of membranes depends on lipid chain saturation. Phys. Rev. E.

[41] Baumgart T., Das S., Webb W.W., Jenkins J.T. (2005). Membrane elasticity in giant vesicles with fluid phase coexistence. Biophys. J..

[42] Rawicz W., Olbrich K.C., McIntosh T., Needham D., Evans E.A. (2000). Effect of chain length and unsaturation on elasticity of lipid bilayers. Biophys. J..

[43] Kalutskii M.A., Galimzyanov T.R., Pinigin K.V. (2023). Determination of elastic parameters of lipid membranes from simulation under varied external pressure. Phys. Rev. E.

[44] Hamm M., Kozlov M.M. (2000). Elastic energy of tilt and bending of fluid membranes. Eur. Phys. J. E.

[45] Nagle J.F. (2017). Experimentally determined tilt and bending moduli of single-component lipid bilayers. Chem. Phys. Lipids.

[46] Morris C.E., Homann U. (2001). Cell surface area regulation and membrane tension. J. Membr. Biol..

[47] Sens P., Plastino J. (2015). Membrane tension and cytoskeleton organization in cell motility. J. Phys. Condens. Matter.

[48] Blosser M.C., Honerkamp-Smith A.R., Han T., Haataja M., Keller S.L. (2015). Transbilayer colocalization of lipid domains explained via measurement of strong coupling parameters. Biophys. J..

[49] Galimzyanov T.R., Kuzmin P.I., Pohl P., Akimov S.A. (2017). Undulations drive domain registration from the two membrane leaflets. Biophys. J..

[50] Kondrashov O.V., Galimzyanov T.R., Jiménez-Munguía I., Batishchev O.V., Akimov S.A. (2019). Membrane-mediated interaction of amphipathic peptides can be described by a one-dimensional approach. Phys. Rev. E.

[51] Braganza L.F., Worcester D.L. (1986). Structural Changes in Lipid Bilayers and Biological Membranes Caused by Hydrostatic Pressure. Biochemistry.

[52] Scarlata S.F. (1991). Compression of lipid membranes as observed at varying membrane positions. Biophys. J..

[53] Tosh R.E., Collings P.J. (1986). High pressure volumetric measurements in dipalmitoylphosphatidylcholine bilayers. Biochim. Biophys. Acta (BBA)-Biomembr..

[54] Vennemann N., Lechner M.D., Henkel T., Knoll W. (1986). Densitometric Characterization of the Main Phase Transition of Dimyristoyl-Phosphatidylcholine between 0.1 and 40 MPa. Berichte Bunsenges. Phys. Chem..

[55] Berger O., Edholm O., Jähnig F. (1997). Molecular dynamics simulations of a fluid bilayer of dipalmitoylphosphatidylcholine at full hydration, constant pressure, and constant temperature. Biophys. J..

[56] Venable R.M., Skibinsky A., Pastor R.W. (2006). Constant surface tension molecular dynamics simulations of lipid bilayers with trehalose. Mol. Simul..

[57] Pinigin K.V. (2022). Determination of Elastic Parameters of Lipid Membranes with Molecular Dynamics: A Review of Approaches and Theoretical Aspects. Membranes.

[58] Tossi A., Sandri L., Giangaspero A. (2000). Amphipathic, α-helical antimicrobial peptides. Biopolymers.

[59] Ludtke S.J., He K., Wu Y., Huang H.W. (1994). Cooperative membrane insertion of magainin correlated with its cytolytic activity. Biochim. Biophys. Acta (BBA)-Biomembr..

[60] Huang H.W. (2006). Molecular mechanism of antimicrobial peptides: The origin of cooperativity. Biochim. Biophys. Acta (BBA)-Biomembr..

[61] Ludtke S., He K., Huang H. (1995). Membrane thinning caused by magainin 2. Biochemistry.

[62] Perrin B.S., Sodt A.J., Cotten M.L., Pastor R.W. (2015). The curvature induction of surface-bound antimicrobial peptides piscidin 1 and piscidin 3 varies with lipid chain length. J. Membr. Biol..

[63] Smith B.J. (2012). PS–a program for the analysis of helix geometry. J. Mol. Graph. Model..

[64] Counterman A.E., Clemmer D.E. (1999). Volumes of individual amino acid residues in gas-phase peptide ions. J. Am. Chem. Soc..

[65] Vitkova V., Méléard P., Pott T., Bivas I. (2006). Alamethicin influence on the membrane bending elasticity. Eur. Biophys. J..

[66] Bouvrais H., Méléard P., Pott T., Jensen K.J., Brask J., Ipsen J.H. (2008). Softening of POPC membranes by magainin. Biophys. Chem..

[67] Shchelokovskyy P., Tristram-Nagle S., Dimova R. (2011). Effect of the HIV-1 fusion peptide on the mechanical properties and leaflet coupling of lipid bilayers. New J. Phys..

[68] Leibler S. (1986). Curvature instability in membranes. J. Phys..

[69] Galimzyanov T.R., Molotkovsky R.J., Bozdaganyan M.E., Cohen F.S., Pohl P., Akimov S.A. (2015). Elastic membrane deformations govern interleaflet coupling of lipid-ordered domains. Phys. Rev. Lett..

[70] Perlmutter J.D., Sachs J.N. (2011). Interleaflet interaction and asymmetry in phase separated lipid bilayers: Molecular dynamics simulations. J. Am. Chem. Soc..

[71] Schäfer L.V., Marrink S.J. (2010). Partitioning of lipids at domain boundaries in model membranes. Biophys. J..

[72] Galimzyanov T.R., Molotkovsky R.J., Kheyfets B.B., Akimov S.A. (2013). Energy of the interaction between membrane lipid domains calculated from splay and tilt deformations. JETP Lett..

[73] Israelachvili J.N. (2011). Intermolecular and Surface Forces.

[74] Verkleij A.J., Zwaal R.F.A., Roelofsen B., Comfurius P., Kastelijn D., Van Deenen L.L.M. (1973). The asymmetric distribution of phospholipids in the human red cell membrane. A combined study using phospholipases and freeze-etch electron microscopy. Biochim. Biophys. Acta (BBA)-Biomembr..

[75] Lorent J.H., Levental K.R., Ganesan L., Rivera-Longsworth G., Sezgin E., Doktorova M., Lyman E., Levental I. (2020). Plasma membranes are asymmetric in lipid unsaturation, packing and protein shape. Nat. Chem. Biol..

[76] Akimov S.A., Aleksandrova V.V., Galimzyanov T.R., Bashkirov P.V., Batishchev O.V. (2017). Interaction of amphipathic peptides mediated by elastic membrane deformations. Biol. Membr..

[77] Li C., Salditt T. (2006). Structure of magainin and alamethicin in model membranes studied by x-ray reflectivity. Biophys. J..

[78] Veatch S.L., Polozov I.V., Gawrisch K., Keller S.L. (2004). Liquid domains in vesicles investigated by NMR and fluorescence microscopy. Biophys. J..

[79] Klauda J.B., Venable R.M., Freites J.A., O’Connor J.W., Tobias D.J., Mondragon-Ramirez C., Vorobyov I., Mackerell Jr A.D., Pastor R.W. (2010). Update of the CHARMM all-atom additive force field for lipids: Validation on six lipid types. J. Phys. Chem. B.

[80] Owen D.M., Magenau A., Williamson D., Gaus K. (2012). The lipid raft hypothesis revisited–new insights on raft composition and function from super-resolution fluorescence microscopy. Bioessays.

[81] Oren Z., Shai Y. (1998). Mode of action of linear amphipathic α-helical antimicrobial peptides. Pept. Sci..

[82] Huan Y., Kong Q., Mou H., Yi H. (2020). Antimicrobial peptides: Classification, design, application and research progress in multiple fields. Front. Microbiol..

